# Rabies Update for Latin America and the Caribbean

**DOI:** 10.3201/eid1904.121482

**Published:** 2013-04

**Authors:** Marco A.N. Vigilato, Ottorino Cosivi, Terezinha Knöbl, Alfonso Clavijo, Hugo M.T. Silva

**Affiliations:** Pan American Health Organization, Rio de Janeiro, Brazil (M.A.N. Vigilato, O. Cosivi, A. Clavijo, H.M.T. Silva);; Universidade de São Paulo, São Paulo, Brazil (T. Knöbl)

**Keywords:** Rabies, rabies virus, viruses, zoonoses, epidemiology, prevention, control, dogs, bats, animals, Latin America, Caribbean

**To the Editor:** Rabies incidence in Latin America and the Caribbean has decreased and several countries (Uruguay, Chile, Costa Rica, Mexico, and Panama) and areas of Peru, Brazil, and Argentina are free of human rabies transmitted by dogs, although there are certain areas to which this disease is still endemic (*1*). Coordinated actions for regional elimination of human rabies transmitted by dogs began in 1983 in Latin America and the Caribbean with the assistance of the Pan American Health Organization (PAHO). This effort has led to an ≈90% reduction of human and canine rabies (*2*). In this region, rabies is associated with poverty and considered a neglected disease (*3*). Resolution 19 of the 49th Directing Council of PAHO in 2009 regarding neglected diseases and other infections related to poverty set a target for eliminating human rabies transmitted by dogs by 2015. PAHO is currently developing strategies to assist countries during this period (*4*).

Since 2010, a total of 111 human rabies cases transmitted by bats, dogs, and other animal species were reported from Latin America and the Caribbean: 40 transmitted by dogs and 63 by bats (Table). Although a major reduction in human rabies transmitted by dogs was observed in 2010 (only 6 cases), the total number of cases increased to 24 in 2011; most were confirmed by laboratory testing.

**Table:** Cases of human rabies in 10 countries in Latin America and the Caribbean, 2010–2012*

Country	Rabies transmitted by other animals				Rabies transmitted by bats				Rabies transmitted by dogs			Total
	2010	2011	2012†		2010	2011	2012†		2010	2011	2012†	
Bolivia	0	0	1‡		0	0	0		0	5	1	7
Brazil	1§	0	2‡§		1	0	1		1	2	2	10
Colombia	3¶	0	0		1	0	0		0	0	0	4
Ecuador	0	0	0		0	12	0		0	0	0	12
Guatemala	0	0	0		0	0	0		0	3	0	3
Haiti	0	0	0		0	0	0		1	13	2	16
Honduras	0	0	0		0	0	0		0	0	1	1
Mexico	0	1#	0		4	2	0		0	0	0	7
Peru	0	0	0		13	19	10		1	1	2	46
Dominican Republic	0	0	0		0	0	0		3	0	2	5
Total	4	1	3		19	33	11		6	24	10	111

*Data were obtained from the Regional Information System of Epidemiologic Surveillance of Rabies in the Americas/Epidemiologic Information System, Pan American Center for Foot-and-Mouth Disease, Pan American Health Organization–World Health Organization, 2012. †Data were updated in December 2012. ‡Human rabies transmitted by undetermined animal species (variant hematophagous bat). §Human rabies transmitted by a marmoset monkey (*Callithrix jacchus*). ¶Human rabies transmitted by a cat (variant nonhematophagous bat). #Human rabies transmitted by a skunk.

The higher risk areas for human rabies transmitted by dogs, for which more collaboration and financial support are urgently needed, are Haiti, Bolivia, Guatemala, Dominican Republic, and parts of Brazil (Maranhão State) and Peru (Puno Region). Unfavorable conditions in which persons in these areas are living limit control strategies and maintain rabies transmission (*3*).

According to the PAHO Epidemiologic Surveillance System for Rabies, during 2010–2012, Bolivia and Haiti had the highest incidence of human rabies transmitted by dogs in the Western Hemisphere: 15% (6/40) and 40% (16/40) of all cases, respectively (*5*). Many factors, including national disasters and social, cultural, and economic factors, have interfered with canine rabies control programs in these countries.

Bolivia has a population of 10 million, and 60.0% of the population is considered below the national poverty line. This country has poor suburbs on the outskirts of large cities, with large populations of unowned dogs and limited resources to implement dog mass vaccination campaigns and animal birth control programs. Haiti has a population of >10 million, and 77% of the population is considered below the national poverty line. In 2010, Haiti was devastated by a major earthquake that affected all sectors, including laboratory diagnosis for rabies (*6*). After the earthquake, the country was struck by a cholera epidemic. Financial resources have been diverted to control such priorities and to provide humanitarian aid. Haiti and Bolivia heavily depend on technical cooperation and donations from other governments or institutions, and are a high priority for elimination of human rabies transmitted by dogs (*7**)*.

Another challenge for Latin America and the Caribbean is development of a common strategy for preventing human rabies transmitted by bats, especially in remote areas in the Amazon region (Peru, Ecuador, and Brazil) and Mexico (*7*), from which 97% of human rabies cases were reported during this period. Since 2000, vampire bats have been the leading cause of human rabies in Latin America and the Caribbean (*8*). Comparison of data for 2010–2012 with data for the previous 3 years shows a 5.2% increase in bat-transmitted human rabies, especially during 2011, which accounted for ≈53% of reports during the past 3 years (*5*).

Bats have been identified as a reservoir for many *Lyssavirus* spp. genotypes, and the geographic distribution of variants has been associated with climate changes and ecologic imbalances. Spread of bats has been facilitated by human-made shelters near human dwellings (*9*).

Although rabies control in Latin America and the Caribbean has been successful, certain approaches currently used, such as mass vaccination campaigns for dogs, postexposure prophylaxis, and epidemiologic surveillance, require improvement in some countries. In addition, allocation of resources is needed to enhance national programs to eliminate human rabies transmitted by dogs.

PAHO is responsible for coordination and technical cooperation of the Rabies Elimination Program and Operation of the Epidemiologic Surveillance System for Rabies. For the past 60 years, the Pan American Center for Foot-and-Mouth Disease/PAHO has accumulated capabilities to develop national programs for zoonoses prevention and control, particularly for rabies elimination in Latin America and the Caribbean.

Strengthening regional, national, and subnational rabies control programs must be a priority. The decision in Latin America and the Caribbean to eliminate dog-transmitted rabies began in 1983 and involved strong political commitment with multinational efforts, as well as support and coordination of other international organizations, nongovernmental organizations, and the private sector. This interinstitutional collaboration is needed to promote prevention and control activities to achieve the elimination of human rabies transmitted by dogs in the Western Hemisphere by 2015.
